# Long-Term Maintenance of Viable Human Endometrial Epithelial Cells to Analyze Estrogen and Progestin Effects

**DOI:** 10.3390/cells13100811

**Published:** 2024-05-09

**Authors:** Muhammad Assad Riaz, Franziska Louisa Kary, Alexandra Jensen, Felix Zeppernick, Ivo Meinhold-Heerlein, Lutz Konrad

**Affiliations:** 1 Institute of Gynecology and Obstetrics, Faculty of Medicine, Justus Liebig University Giessen, 35392 Giessen, Germany; muhammad.a.riaz@gyn.med.uni-giessen.de (M.A.R.); franziska.l.kary@med.uni-giessen.de (F.L.K.); felix.zeppernick@gyn.med.uni-giessen.de (F.Z.); ivo.meinhold-heerlein@gyn.med.uni-giessen.de (I.M.-H.); 2 Institute of Radiooncology and Radiotherapy, Clinic Fulda, 36043 Fulda, Germany; alexandra.jensen@klinikum-fulda.de

**Keywords:** endometrial epithelial cells, tight junctions, estrogens, progesterone, progestin, conditional reprogramming

## Abstract

There are fewer investigations conducted on human primary endometrial epithelial cells (HPEECs) compared to human primary endometrial stromal cells (HPESCs). One of the main reasons is the scarcity of protocols enabling prolonged epithelial cell culture. Even though it is possible to culture HPEECs in 3D over a longer period of time, it is technically demanding. In this study, we successfully established a highly pure, stable, and long-term viable human conditionally reprogrammed endometrial epithelial cell line, designated as eCRC560. These cells stained positive for epithelial markers, estrogen and progesterone receptors, and epithelial cell–cell contacts but negative for stromal and endothelial cell markers. Estradiol (ES) reduced the abundance of ZO-1 in a time- and dose-dependent manner, in contrast to the dose-dependent increase with the progestin dienogest (DNG) when co-cultured with HPESCs. Moreover, ES significantly increased cell viability, cell migration, and invasion of the eCRC560 cells; all these effects were inhibited by pretreatment with DNG. DNG withdrawal led to a significantly disrupted monolayer of eCRC560 cells in co-culture with HPESCs, yet it markedly increased the adhesion of eCRC560 to the human mesothelial MeT-5A cells. The long-term viable eCRC560 cells are suitable for in vitro analysis of HPEECs to study the epithelial compartment of the human endometrium and endometrial pathologies.

## 1. Introduction

The endometrium consists mainly of two different cell types: stromal and epithelial cells [1]. The uterine epithelium is usually considered a unit, although strictly speaking, one should distinguish between the luminal surface epithelium (LE) and the glandular epithelium [1]. Likewise, the epithelial cells of the functionalis should be distinguished from the epithelial cells of the basalis. Nevertheless, the LE differs only slightly from the glandular epithelial cells in ultrastructure. Based upon analysis of the ultrastructure, four human epithelial cell types were identified: (1) Ciliated epithelial cells; (2) microvilli-rich epithelial cells; (3) pinopodes; and (4) vesiculated cells [1]. In contrast, recent single-cell RNA sequencing studies revealed between 1 and 10 different epithelial cell types, with no consensus defined to date [2,3,4,5,6,7,8,9,10]. Only three studies mentioned the LE explicitly [4,6,7].

Endometrial epithelial cells, like other epithelial cells, are characterized by a columnar morphology, apical–basal polarity, lateral junctions forming the epithelial cell sheet, and a barrier [1]. To the best of our knowledge, the first paper on the isolation of HPEECs was published by Ryan et al. [11]. Since then, some improvements or variations have been published regarding the isolation procedure [12,13], cryopreservation [14], and the long-term maintenance of human endometrial epithelial stem cells [15]. However, a severe disadvantage of HPEECs is that they show a reduced or lack of response to ovarian steroid hormones [16].

Recently, several papers have been published in which three-dimensional (3D) cell culture was used to generate long-term viable endometrial epithelial organoids and responsiveness to reproductive hormones was studied, but cultivation as 2D monolayers was not described [17,18,19]. Culture of mouse primary epithelial cells and HPEECs as pure 2D monolayers was achieved with conditional reprogramming (CRP) [20,21]; however, long-term HPEEC propagation was not clearly demonstrated [21]. Interestingly, the culture of HPEECs with stromal feeder cells was reported up to passage five without CRP [22]. 

CRP has now become a successful method for the long-term culture of primary epithelial cells, with very little change in cell properties and maintenance of cell lineage commitment [23,24]. CRP is based on the co-culture of irradiated 3T3 J2 mouse fibroblasts (feeder cells) with primary epithelial cells in the presence of the ROCK inhibitor Y-27632 [25,26]. However, the irradiated feeder cells can also be replaced with a conditioned medium (CM) generated from the irradiated feeder cells [26]. Y-27632 was originally shown to promote human keratinocyte viability [27,28], where it induced CRP and unlimited cell proliferation [29]. As previously shown in keratinocytes, the removal of the CM restored the cells’ ability to differentiate [26]. Although the exact CRP mechanism is still unclear, previous studies have shown that the combination of F-medium with feeder cells and Y-27632 exerts two functions to promote unrestricted cell proliferation: (1) induction of telomerase and (2) cytoskeletal remodeling and/or interference with the p16/retinoblastoma signaling pathway [25,30].

Of all organ epithelia, the human endometrium is unique because it not only forms a physical barrier and confers protection against pathogens [31] but also undergoes remodeling for the implantation of the blastocyst and regenerates without scars about once a month, without functional losses [32,33]. While the stromal cells are reprogrammed for implantation, namely decidualization, the epithelial cells have to loosen the barrier for implantation [32], and most of the functionalis layer is shed in a piecemeal process and renewed during menstruation [34].

So far, the long-term cultivation of HPEECs as 2D monocultures has only rarely been successful. Therefore, our main goal was to overcome the limitations of culturing HPEECs. The described CRP method enables experiments on the involvement of HPEECs/eCRCs in the endometrial epithelial barrier against pathogens, their modification for blastocyst implantation, their contribution to regeneration after menstruation, and the formation of 3D organoids. In addition, research on the involvement of HPEECs/eCRCs in endometrial pathologies (endometriosis, adenomyosis, and endometrial cancer) will be facilitated.

## 2. Material and Methods

This study was approved by the local ethics committee of Justus Liebig University (Ethics Committee of the Department of Medicine, permit number: 95/09, Giessen, Germany). The participants gave preoperatively signed consent for participation in the study. All experiments were performed in accordance with relevant guidelines and regulations.

### 2.1. Cell Culture of Mesothelial Cells

Human mesothelial MeT-5A cells (CRL-9444) were purchased from the American Type Culture Collection (ATCC, Manassas, VA, USA) and routinely cultured in M199 medium supplemented with 5% FCS, 1% non-essential amino acids, and 1% penicillin/streptomycin (pen-strep) at 37 °C and 5% CO_2_. Cell culture reagents were purchased from ThermoFisher Scientific (Karlsruhe, Germany).

### 2.2. Isolation of Human Primary Endometrial Stromal and Epithelial Cells

Human primary endometrial epithelial stromal cells (HPESCs) and human primary endometrial epithelial cells (HPEESCs) were isolated from the eutopic endometrial tissue of a 45-year-old woman who underwent surgery by abdominal total laparoscopic hysterectomy due to endometriosis and uterine leiomyoma. The patient had not received hormonal therapy before surgery. HPEESCs and HPESCs were isolated and cultured as previously described with some modifications [6,35,36].

Briefly, endometrial tissue was minced into small pieces (1–3 mm) in Dulbecco Modified Eagle Medium (DMEM; Gibco, Darmstadt, Germany) supplemented with 10% fetal bovine serum (FCS; Gibco), 1% pen-strep (Thermo Fisher), and incubated with 500 µg/mL clostridium histolyticum collagenase (Sigma-Aldrich, Taufkirchen, Germany) at 37 °C for 2 h in a shaking water bath. After enzymatic digestion, the dispersed tissue fragments were passed through 100 μm cell strainers (Corning/Greiner, Frickenhausen, Germany), resulting in the first filtrate (filtrate-1) and tissue fragments retained on the strainer. The filtrate-1 was centrifuged at 500× *g* for 5 min, and the cell pellet was resuspended in 5 mL DMEM (+10% FCS and 1% pen-strep). The tissue fragments from the cell strainer were transferred to a 50 mL falcon *tube* containing 10 mL of accumax dissociation solution (Innovative Cell Technologies, San Diego, CA, USA) and 10 mL of 0.25% trypsin (Thermo Fisher, Darmstadt, Germany) and incubated for 10 min at room temperature with gentle shaking. After the second enzymatic dissociation, 20 mL of DMEM (+10% FCS) was added to the cell suspension to inhibit trypsin and filtered through a 100 μm cell strainer (Corning/Greiner, Frickenhausen, Germany). This second filtrate (filtrate-2) was centrifuged at 500× *g* for 5 min, and the dissolved pellet was combined with filtrate-1. Additionally, liberated nucleic acids were digested with DNase I (100 µg/mL, Sigma-Aldrich). The combined filtrate-1 and filtrate-2 were centrifuged at 500× *g* for 5 min, and the supernatant was discarded. The pellet was resuspended in DMEM (+4.5 g/L glucose, 10% FCS, 2 mmol/L glutamine, and 1% pen-strep). The endometrial cell suspension contained both epithelial and stromal cells. Then, a differential method was used to separate stromal cells from epithelial cells. Stromal cells, unlike epithelial cells, adhere firmly and quickly to cell culture plates and can then be separated from floating erythrocytes and epithelial cells by plating for some hours [37]. After pre-plating for 18 h in stromal cell culture medium (DMEM/F12 + 2 mmol/L glutamine, 10% FCS, 1% pen-strep, 1% insulin, transferrin, and selenium solution), the supernatant containing the enriched HPEECs was removed and subjected to CRP. The adhered HPESCs were maintained in a stromal cell culture medium in a humidified incubator at 37 °C and 5% CO_2_. The purity of HPESCs was assessed by morphological examination with the stromal markers CD10 (MME), CD248, and the epithelial marker mucin-1 [38]. The purity of HPESCs was greater than 99.5%. HPESCs in passages 4–5 were used for further analysis. The culture media were changed every 48 h for 7–8 days to attain 100% confluence. To propagate HPEECs for the long term, the endometrial cell suspensions were subjected to CRP.

### 2.3. Irradiation of Feeder Cells and Preparation of Conditioned Medium (CM)

The feeder cells (Swiss 3T3 fibroblasts, J2 strain; Kerafast/Biozol, Boston, MA, USA) were cultured as advised by the manufacturer in a humidified incubator at 37 °C and 5% CO_2_. Complete DMEM (+2 mM L-glutamine (Gibco), 10% bovine calf serum (BCS, GE Healthcare/Thermo Fisher), and 1% pen-strep) was replaced every 2–3 days until the feeder cells reached 60–80% confluency in a T175 flask (TPP, Trasadingen, Switzerland). The cells were rinsed with PBS, incubated with TrypLE (Thermo Fisher, Darmstadt, Germany) for 5 min at 37 °C until detachment, and filled up with 10 mL fresh F medium [375 mL complete DMEM + 125 mL F12 nutrient mix (Gibco)]. The cell suspension (1.0–2.5 × 10^6^ cells/mL) was irradiated with a total dose of 30 Gy (=3000 rad) with a Synergy Elekta 6-MV photon linear accelerator, plated in a T175 flask (7.0 × 10^6^ cells/30 mL F medium), and incubated at 37 °C in a humidified incubator with 5% CO_2_. After 72 h, the medium was collected and centrifuged at 300× *g* for 5 min at 4 °C. The supernatant was filtered through 0.22 μm filters (Sigma-Aldrich, St. Louis, MO, USA). For immediate use, one part of the complete F medium was mixed with three parts of CM supplemented with 10 μM ROCK inhibitor (Y-27632diluted in water, Enzo Life Sciences, Farmingdale, NY, USA) and 0.1 nM cholera toxin (C8052; Sigma-Aldrich, diluted in water), resulting in complete CM. The CM was stored at 4 °C for up to 1 week or at −80 °C for the long term.

### 2.4. Induction of CRP in HPEECs with Irradiated Feeder Cells

The supernatant containing the enriched HPEECs was immediately co-cultivated with the irradiated feeder cells 3T3 J2 in complete F medium [3:1 (*v*/*v*) F-12 Nutrient Mixture (Ham)–DMEM (Thermo Fisher), 5% FBS, 5 μg/mL insulin (Sigma-Aldrich), 0.1 nM cholera toxin (diluted in water), 10 ng/mL epidermal growth factor (Invitrogen), and 10 μM of ROCK inhibitor (Y-27632, diluted in water). The co-culture was maintained at 37 °C in a humidified incubator with 5% CO_2_ and passaged when 80–90% confluent.

### 2.5. Separation of Epithelial Cells and Feeder Cells

Differential TrypLE (Thermo Fisher) treatment was used to separate the co-cultured feeder and epithelial cells as described previously with minimal modifications [25,26]. Briefly, co-cultured cells were rinsed with PBS and incubated with TrypLE at room temperature for 1 min under microscopic inspection. When the feeder cells rounded up and dissociated, culture flasks were gently tapped, and detached feeder and stromal cells were carefully aspirated. The epithelial cell colonies remained tightly adhered to the culture plates. The epithelial cell colonies were rinsed again with PBS and again incubated with TrypLE at 37 °C for 5–10 min. After gentle pipetting, the epithelial cells were resuspended in a complete F medium. After centrifugation at 500× *g* for 5 min, the epithelial cell pellets were resuspended in F medium for passaging or in cryopreservative medium for freezing.

### 2.6. Cell Culture and Treatment of Cells with Various Agents

All cell types were cultured in a humidified incubator at 37 °C and 5% CO_2,_ and the medium was routinely renewed every 2–3 days. Cells were washed once with PBS and passaged at about 80% confluence. All cell culture reagents were purchased from Invitrogen/ThermoFisher Scientific (Karlsruhe, Germany) unless stated otherwise. Treatments with hormones were conducted with charcoal-stripped FCS (Gibco, Cat. No. 12676-029). Depending on the scope of the experiment, cells were cultured in 6-well plates or ThinCert cell culture inserts (Greiner, Kremsmünster, Austria) and serum-starved (medium + 1% FCS) for 24 h. Cells were pre-treated in duplicates with either vehicle (ethanol) or dienogest (DNG, 20–100 nM, Sigma-Aldrich) 2 h prior to stimulation with 17β-estradiol (ES, 10–50 nM, Sigma-Aldrich) for various times.

### 2.7. Immunofluorescence (IF)

Immunofluorescence staining was performed as previously reported, with some modifications [39]. A total of 5.0 × 10^4^ eCRC560 cells were plated on 24-well plates (1 mL/well) and incubated at 37 °C and 5% CO_2_ for 48 h. Then, cells were rinsed with PBS, fixed with 100% ice-cold methanol (Roth) on ice for 10 min, and thereafter blocked for 1 h at room temperature in blocking solution (3% BSA, 0.1% Tween 20, 4 × SSC; all from Sigma-Aldrich). The eCRC560 cells were incubated with the primary antibodies in a blocking solution at a 1:300 dilution for the detection of claudin 11 (36-4500; Invitrogen), mucin 1 (SC-7313, Santa Cruz, Santa Cruz, CA, USA), cytokeratin 19 (CK19, NB100-687SS, Novus Biologicals, Wiesbaden Nordenstadt, Germany), estrogen receptor α (ER-α, AB259427, Abcam), progesterone receptor A/B (PgR-A/B, AB16661, Abcam, Cambridge, UK), CD10 (M7308, Agilent DAKO, Santa Clara, CA, USA), and ZO-1 (61-7300; Thermo Fisher) overnight at 4 °C. The secondary antibodies (Alexafluor 488-conjugated donkey anti-rabbit (A-21206), Alexafluor 555-conjugated donkey anti-mouse (A-31570), Alexafluor 555-conjugated donkey anti-rabbit (A-31570), and Alexafluor 488-conjugated donkey anti-mouse secondary antibody (A-21202), all diluted 1:500, and all from Life Technologies/Thermo Fisher) were applied together with 1 µg/mL 4′-,6-diamidino-2-phenylindole (DAPI) nuclear counter-stain for 1 h at room temperature. After washing three times with PBS, images were obtained using an inverse Olympus IX81 (Tokyo, Japan) microscope equipped with a fluorescence system.

### 2.8. Western Blot

Western blot was performed as previously reported, with some modifications [40]. Briefly, epithelial cells were treated with increasing concentrations of ES (10–50 nM) and DNG (20–100 nM) for 24 h and 48 h, and cell lysates were collected. The cell lysates of the endothelial cell line HUVEC were obtained from Dr. Aslam [41]. A total of 20 µg of protein from the cell lysates were loaded onto an SDS-PAGE and blotted onto a PVDF membrane (Merck-Millipore, Darmstadt, Germany) at 0.5 V/cm^2^ for 30 min. The PVDF membrane was blocked with 5% BSA in a Tris-buffered saline 0.5% Tween (TBST) solution for 1 h at room temperature. After blocking with 3% BSA, the membrane was incubated with rabbit anti-ZO-1 (1:1000, 61-7300, Thermo Fisher), rabbit anti-FOXA2 (1:1000, 8186, Cell Signaling Technology), mouse anti-vinculin (1:1000, Sigma-Aldrich, V9264), mouse anti-VE-cadherin (1:1000 sc-9989, Santa Cruz, Santa Cruz, CA, USA), or rabbit anti-GAPDH (1:1000, 2118, Cell Signaling Technology, Danvers, MA, USA) at 4 °C overnight and then incubated with anti-rabbit HRP-conjugated secondary antibody (1:1000, Cell Signaling Technology, 7074) or anti-mouse HRP-conjugated secondary antibody (1:1000, Cell Signaling Technology, 7076) for 1 h at room temperature. Following three washings with TBST buffer for 5 min, the chemiluminescence signal was generated with the SignalFire ECL Kit (Cell Signaling Technology, Danvers, MA, USA) and photographed using the chemiluminescence imaging system Fusion Pulse (Vilber, Eberhardzell, Germany).

### 2.9. Measurement of Transepithelial Electrical Resistance (TEER)

TEER measurement was performed as previously reported [42] with minimal changes. Briefly, eCRC560 cells and HPESCs were co-cultivated in different compartments (insert and well) and remained physically separated. A total of 6.0 × 10^4^ HPESCs/cm^2^ in 800 μL complete F medium were seeded into 24-well plates (Corning, Corning, NY, USA). The HPESCs were allowed to adhere overnight at 37 °C and 5% CO_2_. Subsequently, 24-well inserts (Greiner, Kremsmünster, Austria) with translucent membranes and 0.4 μm pores were placed into the 24-well plate. A total of 200 μL of complete F medium containing 8.0 × 10^4^ eCRC560/cm^2^ were added to each insert, and co-cultures were maintained at 37 °C and 5% CO_2_ for 24 h. Following starvation for 24 h, cells were stimulated with either vehicle control (ethanol), ES (20 nM), DNG (100 nM), or ES along with DNG and further incubated at 37 °C and 5% CO_2_ for 48 h. TEER measurements were conducted with the Millicell ERS-2 epithelial Volt-Ohm meter (Merck Millipore, Burlington, MA, USA). The resistance of cell-free inserts was set to zero, and Ω × cm^2^ was calculated according to the protocol of the manufacturer.

### 2.10. Cell Proliferation, Migration, and Invasion Assays

Epithelial cell viability was assessed by a trypan blue exclusion assay following the manufacturer’s instructions. Briefly, equal numbers of eCRC560 and HPESCs (2 × 10^5^) were co-cultivated for 24 h in different compartments (eCRC560 in the wells; HPESCs on the inserts, 0.4 μm pore size) in 6-well plates (Corning, Corning, Corning, NY, USA). After starvation for 24 h, cells were supplemented with either vehicle control (ethanol), ES (20 nM), DNG (100 nM), or ES along with DNG and further incubated at 37 °C and 5% CO_2_ for 24–48 h. The eCRC560 cells were detached with TrypLE (Thermo Fisher) and counted with the TC20. For the migration and invasion assays, equal numbers of HPEECs and eCRC560 (2 × 10^5^) were co-cultivated and stimulated with ES (20 nM) and DNG (100 nM) in different compartments in 6-well plates as described for cell viability. The eCRC560 cells were detached with TrypLE™ and resuspended in complete CM (+0.1% FCS). A total of 200 μL of the cell suspension (1 × 10^5^ cells for migration and 1.5 × 10^5^ for invasion assay) was added onto a 24-well insert (Greiner) with translucent membranes and 8 μm pores with complete CM containing 10% FBS at the bottom of the wells. ES (20 nM) and DNG (100 nM) were again applied to cells and were present throughout the incubation period. For the invasion assay, the cell culture insert was pre-coated with Matrigel (Corning, Corning, NY, USA, 1:4 diluted with DMEM + 0.1% FCS). After incubation at 37 °C for 24 h, the medium was removed from the inserts, and the upper side of the membrane was wiped with a cotton swab to remove non-migrated and non-invasive cells. The cell culture inserts were placed into a new 24-well plate containing 500 μL of calcein AM (8 μM) in an FCS-free DMEM medium and incubated at 37 °C. After 45 min, the medium in the well was discarded, and cells were again incubated for 10 min at 37 °C in 250 μL of TrypLE™ to detach the cells from the filter. The fluorometric intensity of the eCRC560 cells was measured in a black 96-well plate at 480/520 nm with the Tecan Infinite M200 Elisa plate reader (Tecan, Männedorf, Switzerland). 

Confluent monolayers of epithelial cells were detached using TrypLE (Gibco), washed once with PBS, and suspended in culture medium. To generate epithelial cell spheroids, eCRC560 cells were seeded at densities of 100 and 200 cells per well onto a 24-well spherical plate 5D plate (Kugelmeiers, Erlenbach, Switzerland) and maintained in complete F medium for 24–48 h in a humidified incubator at 37 °C and 5% CO_2_.

### 2.11. Hormone Withdrawal and Endometrial–Mesothelial Cell Attachment Assay

Hormone withdrawal was performed as described previously, with some modifications [43]. Briefly, equal numbers of eCRC560 and HPESCs (2 × 10^5^ each) were co-cultivated for 24 h in different compartments (eCRC560 in wells; HPESCs on inserts, 0.4 μm pore size) in 6-well plates (Corning, Corning, NY, USA). After starvation for 24 h, cells were supplemented with either vehicle control (ethanol) or DNG (100 nM) and incubated at 37 °C and 5% CO_2_. Three treatments were run in parallel: (i) one control in medium with 1% FCS for 5 days; (ii) treatment with 100 nM DNG for 5 days; and (iii) treatment with 100 nM DNG for 2 days, followed by culture in medium for 3 days (hormone withdrawal). Then, cells were harvested and used for the attachment assay.

Attachment of eCRC560 cells to mesothelial MeT-5A cells was performed as described previously with some modifications [44]. In brief, eCRC560 cells were detached with TrypLE labeled with 5µM of calcein-AM, and incubated at 37 °C. After 30 min, 20,000 calcein-AM-labeled eCRC560 cells were added onto confluent mesothelial MeT-5A cells in a 96-well black plate with a complete culture medium containing 1% FBS. After incubation for 2 h at 37 °C, the non-adherent cells were washed away gently with PBS. The fluorometric intensity of the eCRC560 cells was measured at 480/520 nm with the Tecan Infinite M200 Elisa plate reader (Tecan, Männedorf, Switzerland). Each assay was run in a minimum of 8 replicates. 

### 2.12. Statistical Analysis

All experiments were repeated independently at least three times with duplicates. The means and SEM values of all experiments were used for analysis. Comparisons of the means between more than two groups were performed by one-way analysis of variance (ANOVA), followed by Dunnett’s multiple comparison test. Student’s *t*-test was used for comparison of two groups using GraphPad Prism software (Version 5.0, GraphPad Inc., Boston, MA, USA). *p* ≤ 0.05 was considered significant.

## 3. Results

**Figure 1:**
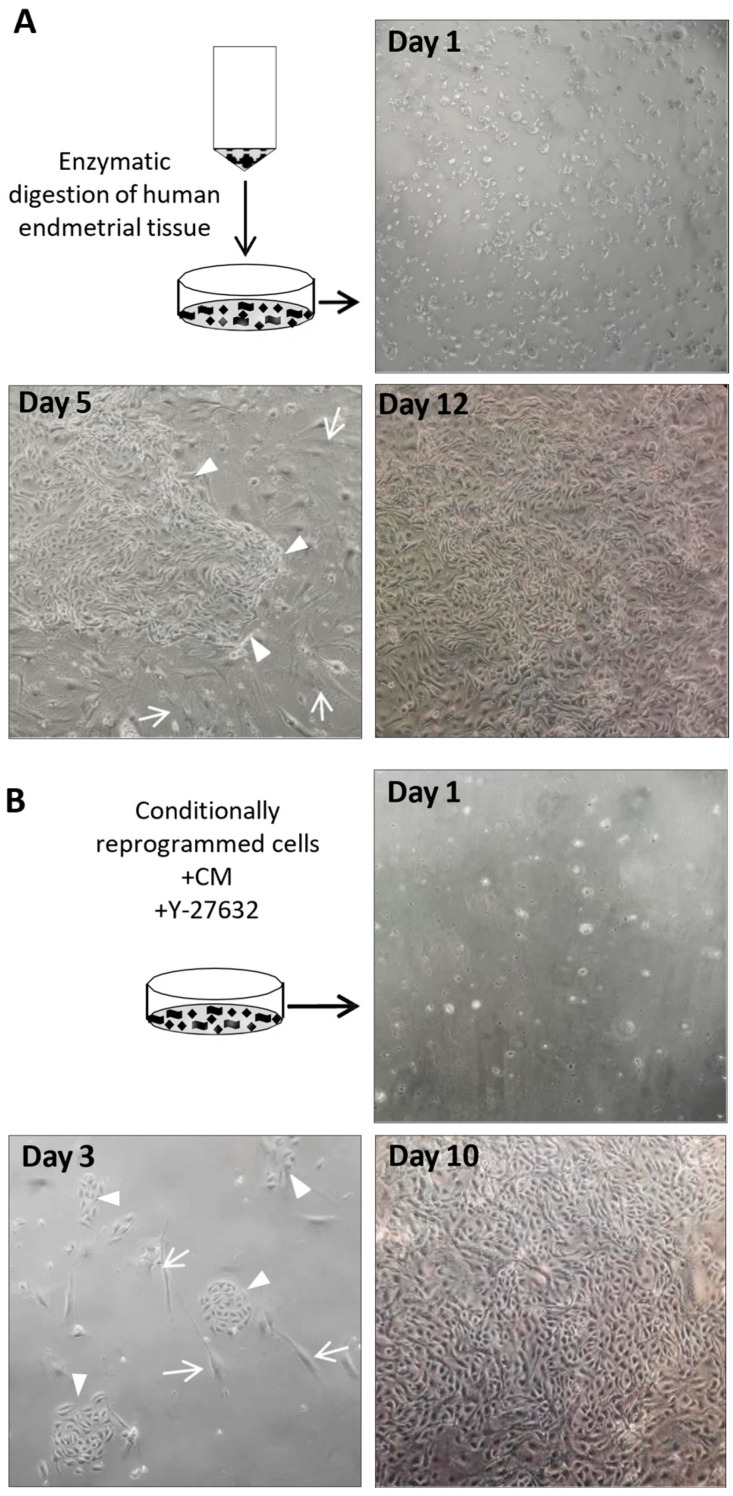
After enzymatic digestion, the cell suspensions were seeded on collagen-coated T25 flasks with irradiated feeder cells in a complete F medium supplemented with the ROCK inhibitor Y-27632. Thereafter, the epithelial cells formed large colonies (arrowheads) with a concomitant decline of the stromal cells (arrows). After 5 days, HPEECs formed colonies that progressed to cell islands the next two days and rapidly proliferated to reach confluence after 12 days (**A**). The conditionally reprogrammed eCRC560 cells were detached after passage four and cultivated in complete CM with the ROCK inhibitor Y-27632. Epithelial cell colonies readily formed after 3 days (arrowheads) with the concomitant death of the stromal cells (arrows). After 10 days, a monolayer of mainly epithelial cells has been formed (**B**). (magnification 100×).

**Figure 2:**
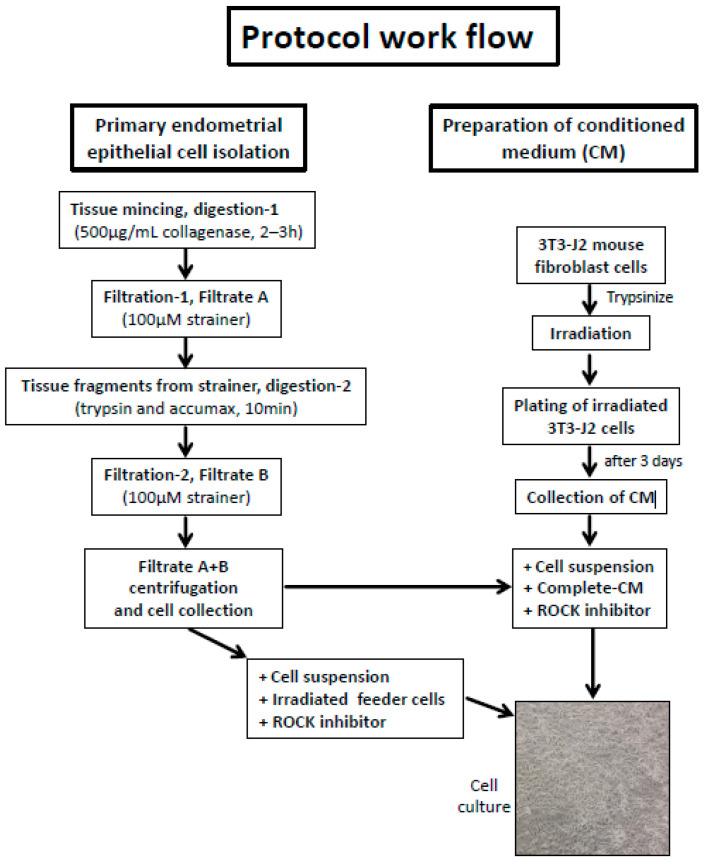
An overview of the CRP protocol, showing mincing and enzymatic digestion of clinical specimens, processing of feeder cells, irradiation, and establishment of co-cultures. CM, conditioned medium.

Small endometrial fragments were dispersed by enzymatic digestion and propagated on irradiated feeder cells in a medium containing the ROCK inhibitor Y-27632 (Figure 1A). The epithelial eCRC560 cells formed small colonies after five days and rapidly proliferated to reach confluence after 12 days (Figure 1A). The irradiated feeder cells inhibited the growth of the stromal cells and supported epithelial cell growth. The epithelial cell colonies were separated by different detachment periods after reaching 80 to 90% confluence and further passage at 1:4 dilutions. After three passages, feeder cells were replaced by a CM medium to establish conditionally reprogrammed cultures using standard culture conditions [30] and to eliminate the need to constantly irradiate feeder cells (Figure 1B). After passage four, the eCRC560 cells were detached and cultivated in complete CM with the ROCK inhibitor Y-27632. Epithelial cell colonies formed after 3 to 5 days with the concomitant death of the stromal cells. After 10 days, a monolayer of mainly epithelial cells has been formed (Figure 1B). After 3–4 passages, pure eCRC560 cells were obtained. A workflow of the CRP protocol is presented in Figure 2.

**Figure 3:**
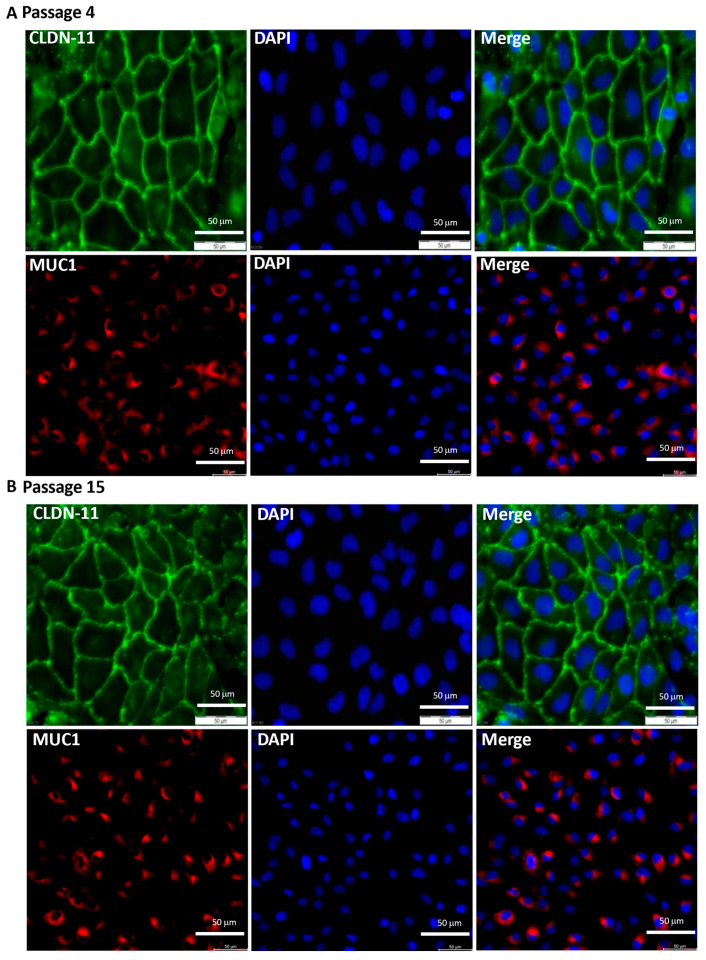
The eCRC560 cells were cultivated in complete CM, and analysis of endometrial epithelial cell markers was performed in early passages ((**A**); passage 4) and in later passages ((**B**), passage 15) by staining with antibodies against claudin-11 (CLDN-11) and mucin-1 (MUC1). Positive staining for claudin 11 (green) and mucin-1 (red) characterized the endometrial epithelial cells. These cells retained their epithelial characteristics even after long-term culture (**B**). Counter-staining was conducted with DAPI (blue), scale bars: 50 µm.

The characterization of the eCRC560 at passage four showed the typical cobblestone-like appearance (Figure 3A,B). Furthermore, all epithelial cells were clearly positive for the endometrial epithelial markers claudin-11 (Cld11) and mucin-1 (MUC1) (Figure 3A,B). Exactly the same morphological and protein pattern was evident in passage 15, indicating that the eCRC560s can proliferate over a long period of time without losing their epithelial phenotype (Figure 3B). Both passages were negative for the endothelial marker VE-Cadherin (Figure S1). In addition, we further detected the high expression of the uterine glandular epithelial-specific marker FOXA2 in the eCRC560s (Figure S2).

**Figure 4:**
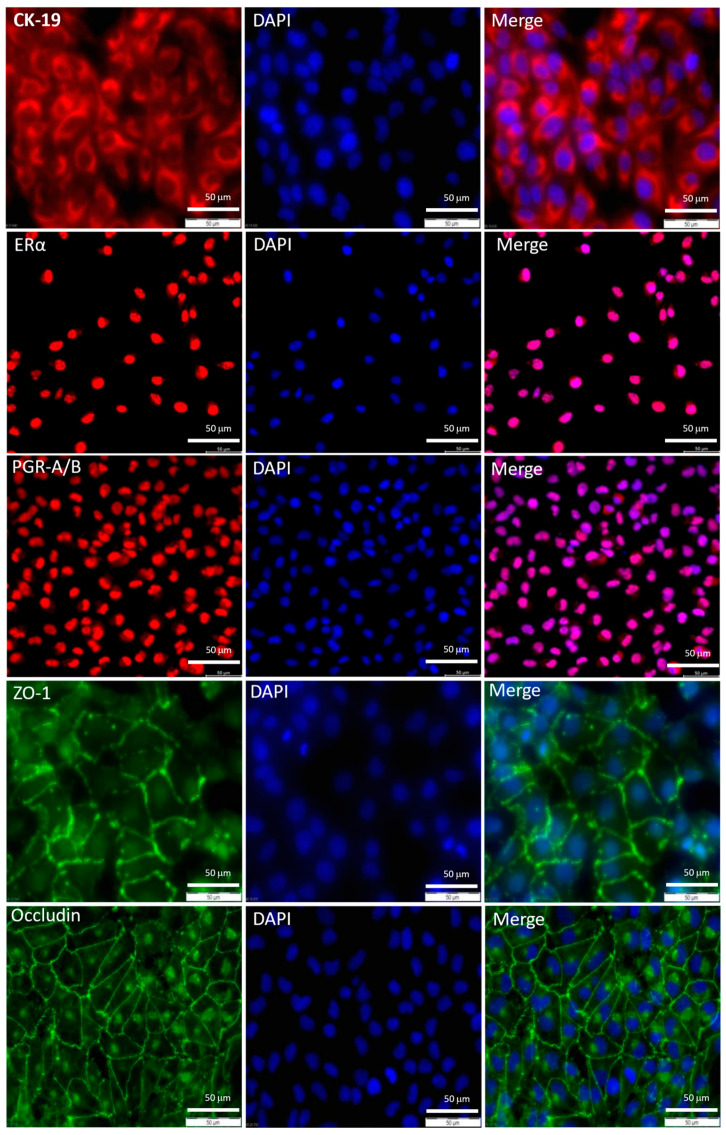
Expression of typical endometrial epithelial cell markers in eCRC560 cells. Expression of endometrial epithelial cell markers, i.e., cytokeratin-19 (CK-19, red), estrogen receptor-α (ERα, green), progesterone receptor (PgR-A/B, red), ZO-1 (green), and occludin (green), was sustained over the course of time (passage 14), suggesting that the epithelial phenotype of the eCRC560 cells is maintained during in vitro culture. Counter-staining was conducted with DAPI (blue); scale bars: 50 µm.

The eCRC560 cells were positive for cytokeratin 19 (Figure 4, CK-19), a marker of human endometrial epithelial cells [38]. Furthermore, examination of the two most important endometrial hormone receptors, whose expression is normally lost after immortalization [9], showed positivity for the estrogen receptor alpha (ERα) and the progesterone receptor A/B (Figure 4, PGR-A/B). Also, the cell–cell contact proteins ZO-1 and occludin were detected in eCRC560 cells (Figure 4). Of note, in addition to the membrane localization, occludin was also found in the nucleus.

For the functional characterization, we investigated the influence of the steroid hormones ES and DNG on cell–cell contacts, cell proliferation, migration, and invasion of the eCRC560 cells. We decided to use the synthetic progestin DNG because it is more stable in cell culture compared to progesterone and is highly selective for the PGR [45]. 

**Figure 5:**
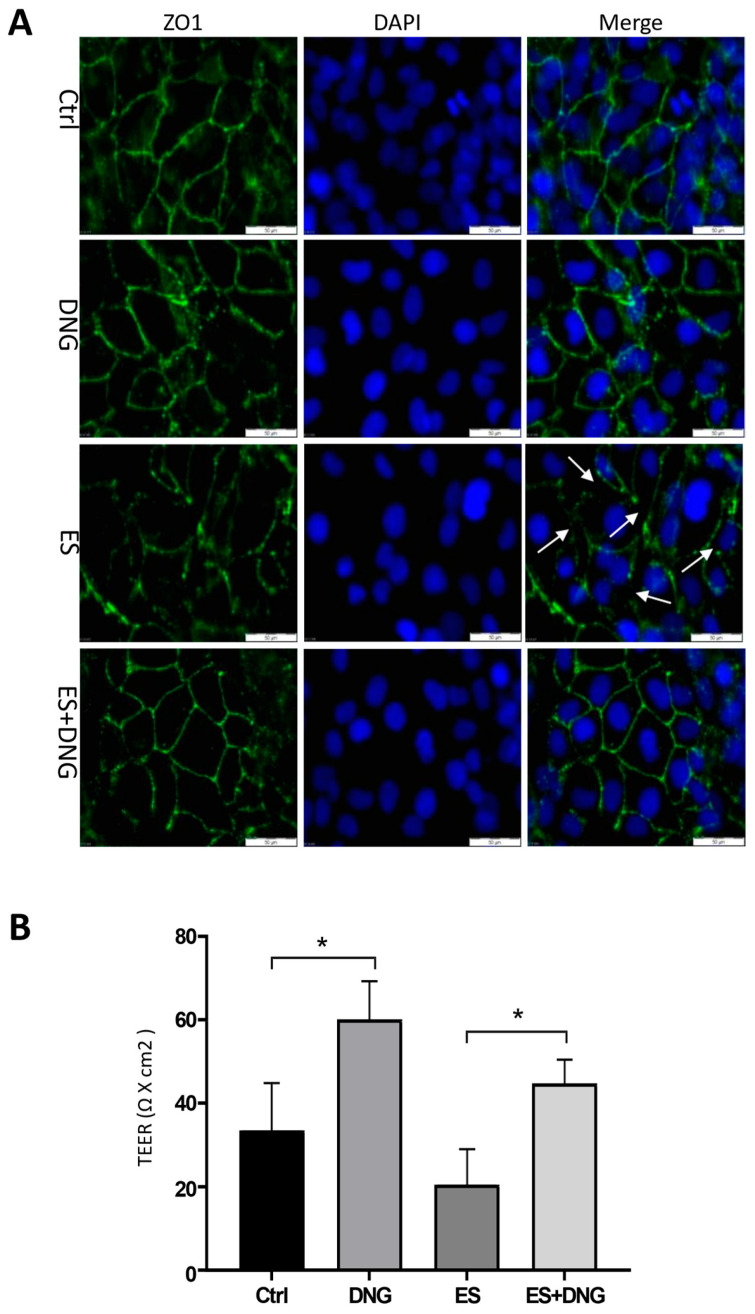
Effects of the ovarian hormones on the localization of ZO-1 in eCRC560 (**A**). Cells were seeded in 24-well plates for 24 h in an indirect co-culture with HPESCs (on the inserts). Localization of ZO-1 (green) was found at the cell–cell contacts. Treatment with ES resulted in the formation of gaps, as exemplified in one slide (arrowheads). Counter-staining was conducted with DAPI (blue); scale bars: 50 µm. Effects of ES and DNG on TEER measurement (**B**). A total of 6.0 × 10^4^ HPESCs/cm^2^ (in the wells) and 8.0 × 10^4^ eCRC560/cm^2^ (on inserts with translucent membranes and 0.4 μm pores) were cultured in 24-well plates for 24 h. TEER measurement showed an increase with DNG but a decrease with ES; co-stimulation showed a mutual effect. Each bar represents the mean ± SEM of three independent experiments performed in duplicate. Dunnett’s test was used for statistical analysis; * *p* ≤ 0.05.

**Figure 6:**
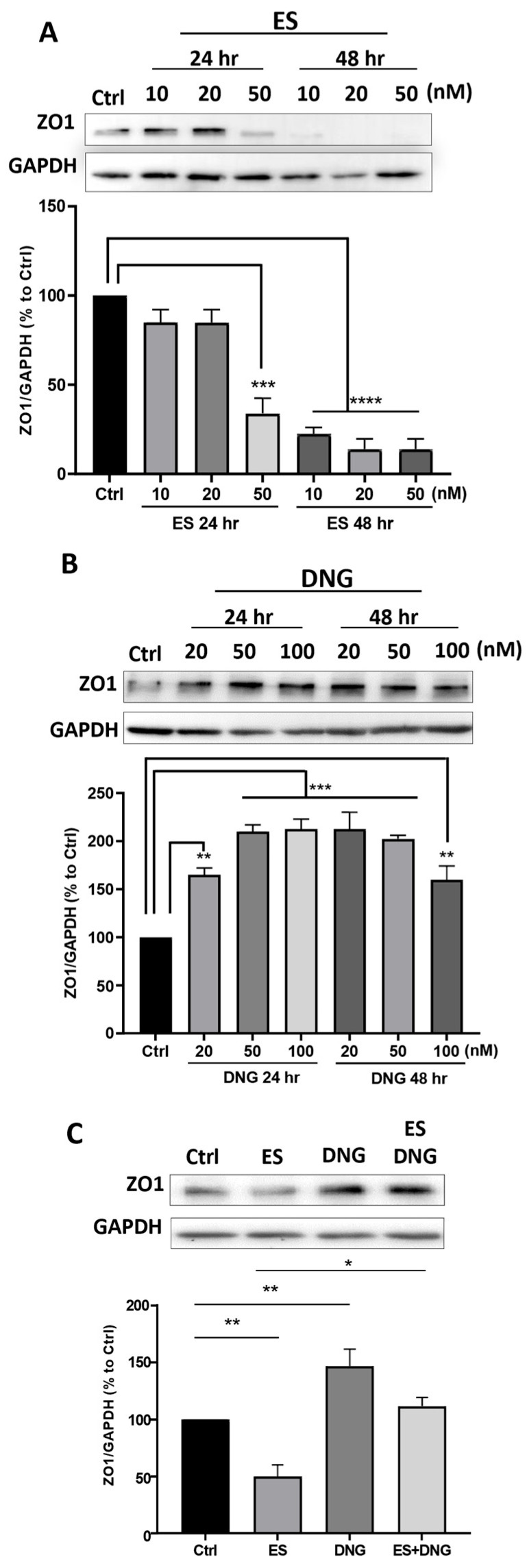
Effects of ES and DNG on ZO1 protein expression in eCRC560 (**A**–**C**). Cells were seeded in 6-well plates for 24 h in an indirect co-culture with HPESCs (on the inserts). ES reduced ZO1 protein levels significantly (**A**), whereas DNG increased ZO1 levels significantly (**B**). Co-stimulation showed that DNG inhibited ES-dependently decreased ZO1 protein abundance (**C**). Each experiment was repeated three times in duplicate and presented as the mean ± SEM. Dunnett’s test was used for statistical analysis; * *p* ≤ 0.05, ** *p* < 0.01, ***p < 0.001, **** *p* < 0.0001.

One of the main regulators of the epithelial barrier is ZO-1, which controls paracellular permeability [46]. The addition of ES to CREC560 resulted in loss of ZO-1 at the cell periphery and induced gaps compared to the control (Figure 5A, arrowheads). DNG treatment promoted the redistribution of ZO-1 to the cell periphery and prevented the ES-induced occurrence of gaps (Figure 5A). TEER measurements, which are a proxy of epithelial tightness, showed an increase with DNG stimulation but a slight decrease with ES alone, which was significantly blocked by co-stimulation with DNG (Figure 5B). Furthermore, stimulation of eCRC560 cells with ES significantly decreased time- and dose-dependently ZO-1 protein levels by ~65% and 75–85% after 24 h and 48 h, respectively (Figure 6A). In contrast, DNG dose-dependently increased ZO-1 levels after 24 h (Figure 6B). A plateau phase was evident after 48 h, except for the highest concentration, which showed a decrease. Moreover, this ES response was prevented by co-incubation of eCRC560 cells with DNG (Figure 6C).

**Figure 7:**
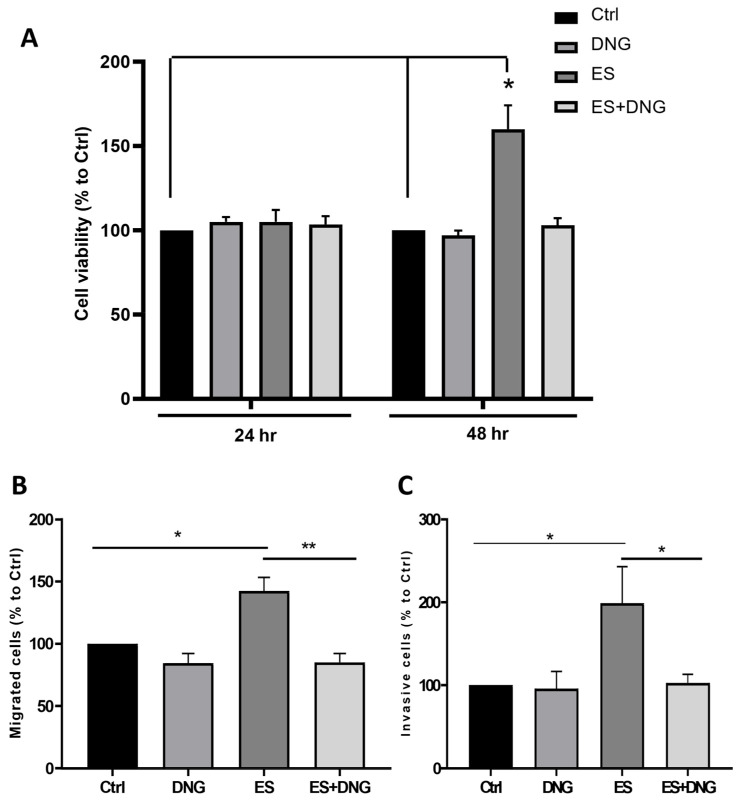
Effects of ES and DNG on epithelial cell viability (**A**), migration (**B**), and invasion (**C**) of eCRC560. Epithelial cells and stromal cells were co-cultivated for 24 h in different compartments in 6-well plates. Only ES significantly increased cell viability after 48 h, which was blocked by DNG (**A**). ES- and DNG-treated cells were plated onto uncoated (cell migration) or Matrigel-coated (cell invasion) inserts (8 μm pores) with complete CM containing 10% FBS at the bottom of a 24-well plate and stimulated for 24 h. Percent cell invasion (**B**) and cell migration (**C**) were calculated as the number of cells that migrated through the uncoated or Matrigel-coated membranes, respectively. ES significantly increased cell migration (**B**) and invasion (**C**) as compared to the untreated control (Ctrl). DNG suppressed ES-dependent increased migration (**B**) and invasion (**C**). Each bar represents the mean ± SEM of 3 independent experiments performed in duplicates. Dunnett’s test was used for statistical analysis; * *p* ≤ 0.05, ** *p* < 0.01.

No effects of ES and DNG alone or in combination on epithelial cell viability were detectable after 24 h (Figure 7A). However, after 48 h, we found a positive effect of ES on epithelial cell viability that was prevented by DNG in the indirect co-culture with HPESCs (Figure 7A). We also observed that ES promoted the migration and invasion of eCRC560, which was significantly suppressed when cells were pre-stimulated with DNG (Figure 7B,C).

**Figure 8:**
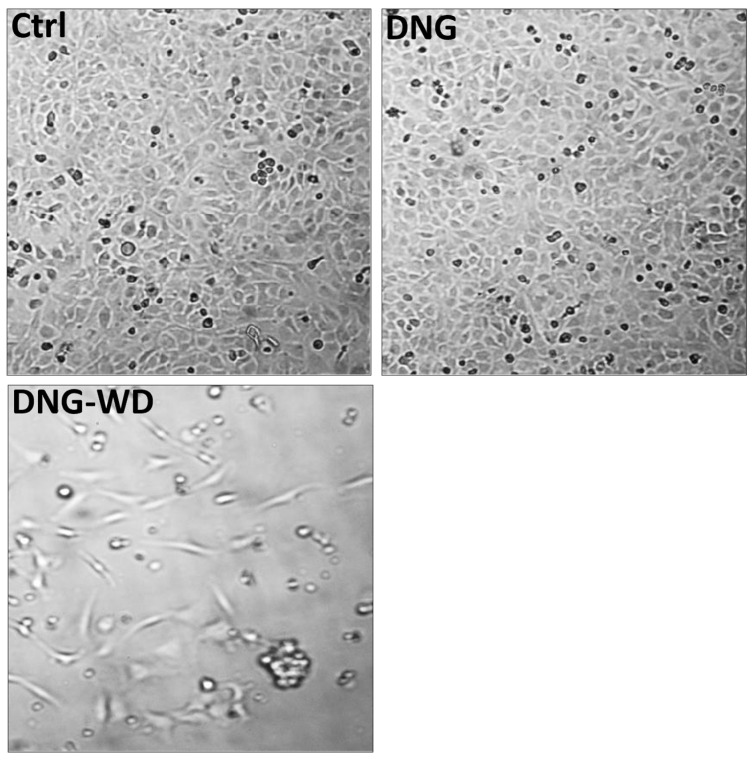
Effects of hormone withdrawal on the monolayers of eCRC560 cells co-cultured with HPESCs. Equal numbers (2.0 × 10^5^) of eCRC560 (in the wells) and HPESCs (on inserts with translucent membranes and 0.4 μm pores) were cultured in 6-well plates for 24 h. DNG withdrawal resulted in a disrupted epithelial monolayer. (magnification 100×).

We used an in vitro system consisting of an indirect co-culture of stromal and the eCRC560 cells to study menstrual breakdown. The integrity of the eCRC560 monolayer was not disturbed in the untreated controls (Ctrl) and DNG-treated cells (Figure 8A,B). However, DNG withdrawal (DNG-WD) induced a strong disruption of the epithelial monolayer integrity (Figure 8C).

**Figure 9:**
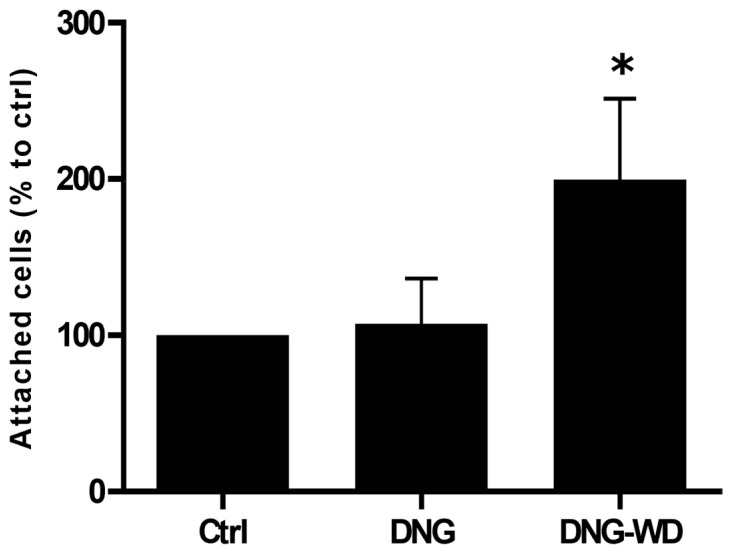
Effects of hormone withdrawal on adhesion of eCRC560 cells to the mesothelial MeT-5A cells. Cells were treated without DNG (Ctrl, ethanol), with 100 nM DNG, and with hormone withdrawal (DNG-WD), as described in Figure 8. Only DNG withdrawal (DNG-WD) induced an increased adhesion of the eCRC560 cells to the mesothelial cells. Each bar represents the mean ± SEM of three independent experiments performed in duplicate. Dunnett’s test was used for statistical analysis; * *p* ≤ 0.05.

Furthermore, we also investigated the cell adhesion of eCRC560 to human mesothelial cells. The adhesion ratio of eCRC560 cells to mesothelial MeT-5A cells was similar in the untreated controls and DNG-treated cells. However, DNG withdrawal (DNG-WD) strongly and significantly increased the attachment of the eCRC560 to the mesothelial cells (Figure 9).

## 4. Discussion

In this study, we used conditional reprogramming for the isolation and long-term maintenance of HPEECs based on Liu et al. [25,26]. The eCRC560 cells were highly pure and viable and retained their epithelial characteristics even after long-term culture. In the beginning, we used irradiated 3T3 J2 feeder cells for CRP of HPEECs but then found that feeder cells can be replaced with the CM from the irradiated feeders using standard culture conditions similar to recent observations with other epithelial cell types [26,30]. The use of CM medium, in contrast to irradiated feeder cells, is more convenient because it eliminates the need to constantly cultivate, maintain, and irradiate feeder cells.

The eCRC560 maintained a cobblestone morphology and expressed typical epithelial proteins like mucin-1, keratin-19, FOXA2, cell–cell contact proteins like claudin-11, occludin, and ZO-1, and most importantly, the steroid hormone receptors ER1 and PGRA/B. Expression of claudin-11 and mucin-1 was retained even after long-term culture. 

So far, there have been several successful attempts to cultivate endometrial epithelial cells, mainly in 3D culture [17,18,19]. All three publications showed a hormonal responsiveness of the organoids to estrogen and progesterone, but only Turco et al. [18] found the expression of the corresponding receptors, albeit only in some but not in all cells. Boretto et al. [17] also detected the ERα only in some cells but did not examine the PGR. Expression of the hormone receptors in only a few epithelial cells in the 3D cultures suggests down-regulation in culture [17,18]. The other studies [20,22] with the culture of HPEECs, with one exception [21], also described the expression of ERα and PGR. In the present study, we have detected ERα and PGR expression in nearly all eCRC560 cells. In vivo, in the proliferative phase, ~95–100% of the glands express ERα and PGR-A, with a decline in the early secretory phase or mid-secretory phase, respectively [47]. 

A more in-depth analysis of the cell–cell contacts and hormone responsiveness revealed that ES decreased, but DNG increased the TEER values of eCRC560 cells in co-culture with stromal cells. Co-stimulation with ES and DNG showed that DNG counteracted ES. These results clearly indicate that eCRC560 cells are hormonally responsive and that ES/DNG regulates human uterine epithelial barrier function. Similarly, it was reported that mouse uterine epithelial monolayer integrity (i.e., TEER) was significantly impaired by estrogen, and estrogen receptors were mediated by matrix metalloproteinases. However, DNG had no effect on ES-induced decreases in the TEER [48]. This lack of the antagonistic effect of DNG might be due to the absence of underlying stromal cells that are required to mediate the effects of progesterone [49]. In contrast, porcine uterine epithelial monolayers were unresponsive to estradiol or progesterone treatment with respect to TEER values [50].

Tight junctions (TJs) are continuous strands that form the primary physical barrier by providing cell–cell contacts in the epithelium. ZO-1 confers adhesiveness to TJs and adherens junctions (AJs), and depletion of ZO-1 results in a defective epithelial barrier for solutes and increased paracellular permeability, accompanied by reorganization of apical actin and myosin [46]. Our studies indicate that stimulation of eCRC560 with ES resulted in a significant dose- and time-dependent decrease in ZO-1 protein as opposed to the positive influence of DNG. Similarly, Jiménez-Salazar et al. [51] have found that estrogen causes ZO-1 disruption in human breast cancer cells. In the human endometrium, ZO-1 is localized at the subapical position of endometrial epithelial cells without any change in localization at the implantation window [52]. Remarkably, human endometrial epithelial cells grown on polyacrylamide gel substrates undergo a partial epithelial-mesenchymal transition with decreased expression of E-cadherin and ZO-1; however, the cells retain their cell–cell contacts [53]. 

TJs, including claudins, occludin, and TJ-associated proteins like ZO-1, are the most apical components of junctional complexes mediating cell–cell contacts in epithelial and endothelial cells. During cellular polarization, occludin gradually accumulates at the ZO-1-positive spot-like junctions to form belt-like TJs in epithelial cells in vitro [54,55]. A direct interaction of occludin and ZO-1 occurs in tight junctions [55]. In the absence of ZO-1, proliferation was increased in intestinal cells, as reviewed in [56], pointing to the role of TJs in cell proliferation [57]. Furthermore, shuttling of ZO-1 to the nucleus was shown to regulate gene expression [58] and to transmit information about the degree of cell–cell contacts to the nucleus to maintain a balance between proliferation and differentiation [57]. Thus, we suggest that the ES-dependent reduction in ZO-1 expression we observed may also be related to cell proliferation.

In accordance with our observation of nuclear localization of occludin, it was also found in astrocytes [59], in epithelial intestinal cells [60], and in cystic fibrosis airway epithelial cells [61]. Localization of occludin to the centrosomes of canine kidney cells was shown to modify mitotic entry [62] or regulate cell proliferation and migration [57]. 

In the current study, we found a positive effect of ES on epithelial cell viability in indirect co-culture with human endometrial stromal cells but not in epithelial monoculture, suggesting that endometrial stromal cells are required to transmit the ES-dependent signal to the endometrial epithelial cells. This ES-dependent effect was expected because, in the endometrium, stromal cells influence the proliferation of endometrial epithelial cells via growth factors like insulin-like growth factor I [63]. 

Much evidence has accrued over the past 50 years on the strong association between progesterone withdrawal and the regulation of menstrual bleeding. The fall in circulating progesterone initiates a sequence of events that ultimately lead to the shedding of the endometrium and menstrual bleeding [43]. To develop an in vitro model of menstrual breakdown, we cultured eCRC560 and HPESCs in an indirect co-culture system simulating progestin withdrawal. Progestin withdrawal induced a strong disruption of the epithelial monolayer integrity, in contrast to the retained epithelial integrity of the untreated controls and progestin-treated cells.

In our study of some steps leading to endometriosis, such as migration, adhesion, and invasion with our epithelial cells, we could support earlier findings that also found a positive effect of ES [64]. To the best of our knowledge, we showed for the first time that the progestin DNG inhibits ES-increased migration and invasiveness of HPEECs. This finding is new, allows a detailed investigation of the mechanisms involved, and is also therapeutically of interest, e.g., to inhibit the initiation of endometriotic lesions. 

Previous studies demonstrated that endometrial cells and fragments rapidly attach to the peritoneal mesothelium [65,66,67]. In the present study, human endometrial epithelial cells were used to evaluate the initial attachment to human mesothelial cells in an in vitro model. Of note, we found that more eCRC560 adhered to mesothelial cells after DNG withdrawal compared to controls or DNG-treated cells. We hypothesize that progesterone deprivation induces MMP expression, which is normally suppressed by progesterone [68]. In particular, inhibition of MMPs-1, -2, -3, -7, and -13 significantly prevented the formation of endometriosis lesions in an in vitro model with chorioallantoic membranes [69].

Three-dimensional in vitro models have attracted much attention in recent years because they can bridge the gap between in vivo and in vitro models and because they closely resemble the original tissues. There is a need for 3D in vitro models to uncover the underlying mechanisms of interactions between cell types and the microenvironment to understand the development and progression of endometriosis [70]. In the present study, we used eCRC560 cells to generate a new but still preliminary in vitro 3D model of the human endometrium in the form of spheroids (Figure S3). The spheroids form very quickly, and no complicated medium change is required, as with the hanging drop technique. The combination of epithelial and stromal cells to generate endometrial spheroids will be investigated in more detail in future experiments.

The isolation of primary epithelial cells enables, among other things, the elucidation of progesterone resistance, as we are currently also attempting to isolate HPEECs from lesions. Flores et al. [71] showed that patients who responded poorly to progestin therapy had reduced PGR expression. The CR method enables not only the long-term cultivation of HPEECs but also the precise elucidation of the reduced receptor expression. Research into pathways such as receptor methylation can be carried out more easily in a 2D rather than a 3D culture. This would be of interest not only diagnostically but also therapeutically. One possible idea would be to restore PGR responsiveness, which would then enable patients to respond better to progestin therapy [72].

## 5. Strength and Limitations

The long-term cultivation of endometrial epithelial cells enables the investigation of individual endometriosis patients over a long period of time so that epithelial cell databases can be established. The endometrial epithelial cells are suitable for hormone studies as well as for pathway analyses, investigations of stroma-epithelial interaction, the production of organoids, and other types of in vitro models. Although we have only presented one epithelial cell line in this publication, we were able to identify two additional lines from endometrium with and without endometriosis, which are currently being characterized in more detail. However, it was important for us to present the successful application of the method as soon as possible. Even though we have not yet found any obvious changes in the endometrial epithelial cells cultured in the long term, we cannot rule out the possibility that there are at least some changes, but this still needs to be investigated using single-cell RNA sequencing or array analysis.

## 6. Conclusions

In this study, we developed a robust protocol that allows highly efficient isolation of primary human epithelial cells from endometrial tissues and successful long-term culture of epithelial cells as monolayers as well as 3D spheroids. Almost all cells expressed the ovarian hormone receptors, maintained their hormone dependency, and showed typical epithelial cell characteristics. Furthermore, we were able to demonstrate the influence of ovarian steroid hormones on ZO-1 protein expression, the epithelial cell barrier, cell migration, and cell invasion, as well as adhesion to mesothelial cells. This strongly suggests the contribution of ovarian steroids to controlling these processes at different phases of the menstrual cycle. The underlying mechanisms by which steroid hormones regulate these events remain to be elucidated. The 2D and 3D endometrial epithelial cell-based model used in the present study will help to understand the process of menstruation and endometriosis and elucidate the underlying mechanisms in detail. This is a breakthrough for all types of in vitro models of the human endometrium and endometrium-related diseases.

## Data Availability

The raw data are available from the corresponding author upon request.

## Supplementary Materials

The following supporting information can be downloaded at https://www.mdpi.com/article/10.3390/cells13100811/s1, Figure S1: Expression of the endothelial marker VE-Cadherin in cell cultures, Figure S2: Expression of the uterine glandular epithelial specific marker FOXA2 in cell cultures, Figure S3: Human endometrial epithelial spheroids.
