# Supplementary material for: Long-Term Maintenance of Viable Human Endometrial Epithelial Cells to Analyze Estrogen and Progestin Effects
Source: Cells. 2024 May 9;13(10):811. doi: 10.3390/cells13100811 (PMC11120542; doi:10.3390/cells13100811)
Supplement: Supplementary file 1 [file cells-13-00811-s001.zip › cells-2949845-supplementary.pdf]

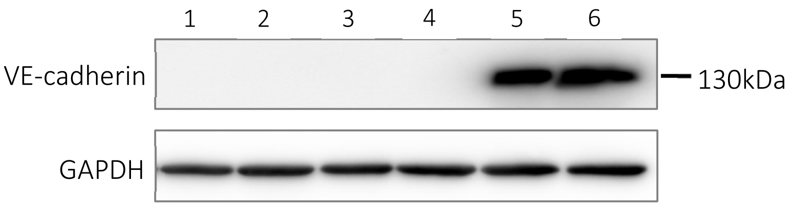

Figure S1 Expression of the endothelial marker VE-Cadherin in cell cultures. Representative western blot for eCRC560 (Lane 1-4) and endothelial HUVECs (Lane 5-6) incubated with antibodies against VE-cadherin. Proteins were extracted from eCRC560 cells at passage 4 (Lane 1-2) and passage 15 (Lane 3-4). Anti-GAPDH was used as an internal control.

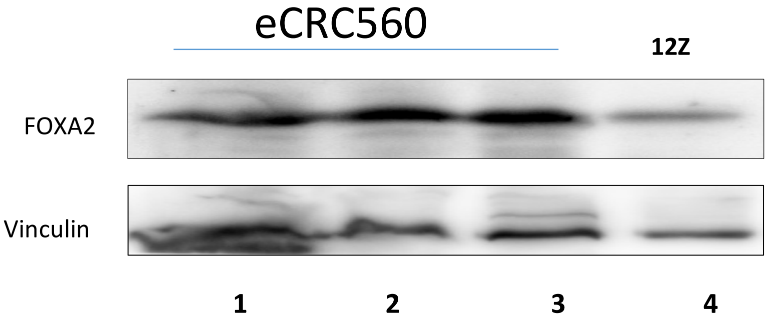

Figure S2 Expression of the uterine glandular epithelial specific marker FOXA2 in cell cultures. Representative western blot for eCRC560 (Lane 1-3) and Immortalized human endometriotic *epithelial cells* (Lane 4) incubated with antibodies against FOXA2. Proteins were extracted from eCRC560 cells at passage 4 (Lane 1-2) and passage 15 (Lane 3). Anti-vinculin was used as an internal control

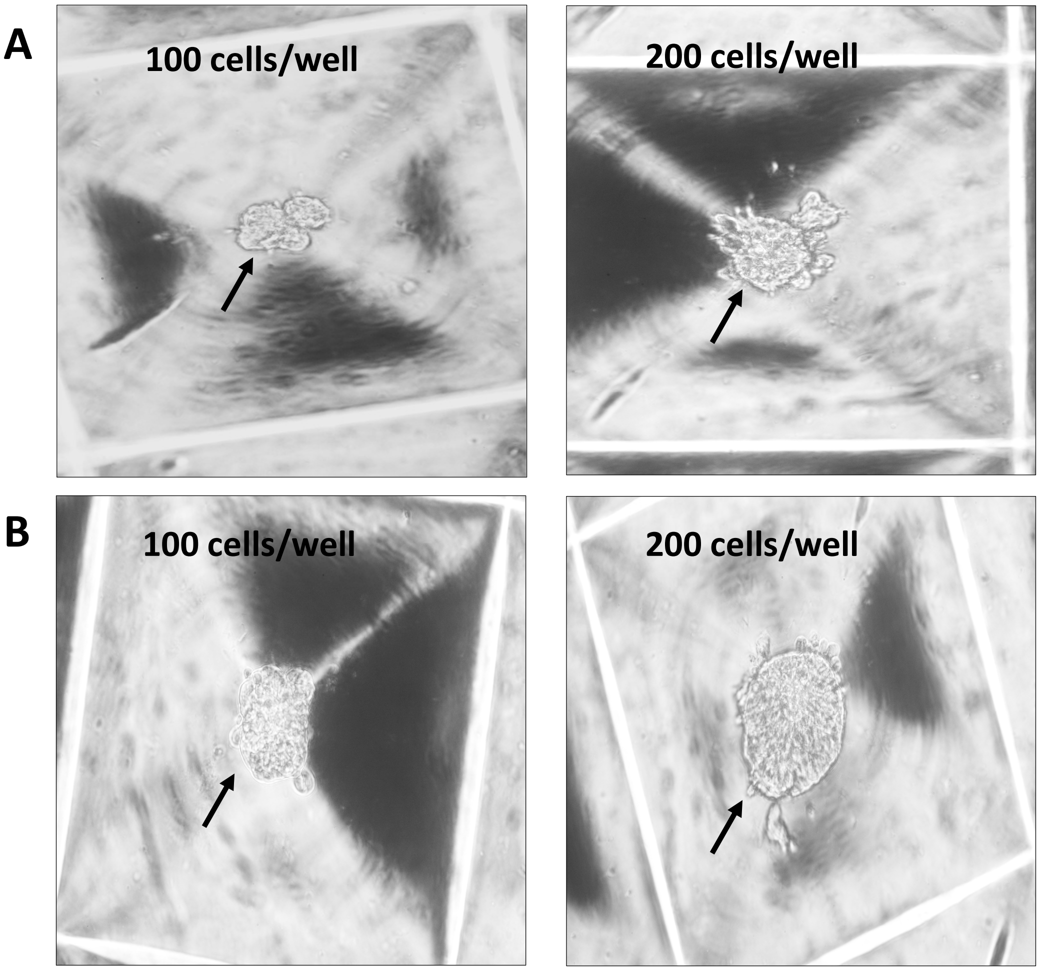

Figure S3 Human endometrial epithelial spheroids. Phase-contrast microscopic images of spheroids (arrows) generated after 24 hr (A) and 48 hr (B) in the Sphericalplate 5D. Magnification: 20x.
